# Supplementary material for: Geographic variation and core microbiota composition of Anastrepha ludens (Diptera: Tephritidae) infesting a single host across latitudinal and altitudinal gradients
Source: PeerJ. 2024 Dec 13;12:e18555. doi: 10.7717/peerj.18555 (PMC11648694; doi:10.7717/peerj.18555)
Supplement: Supplemental Information 1 [file peerj-12-18555-s001.docx]

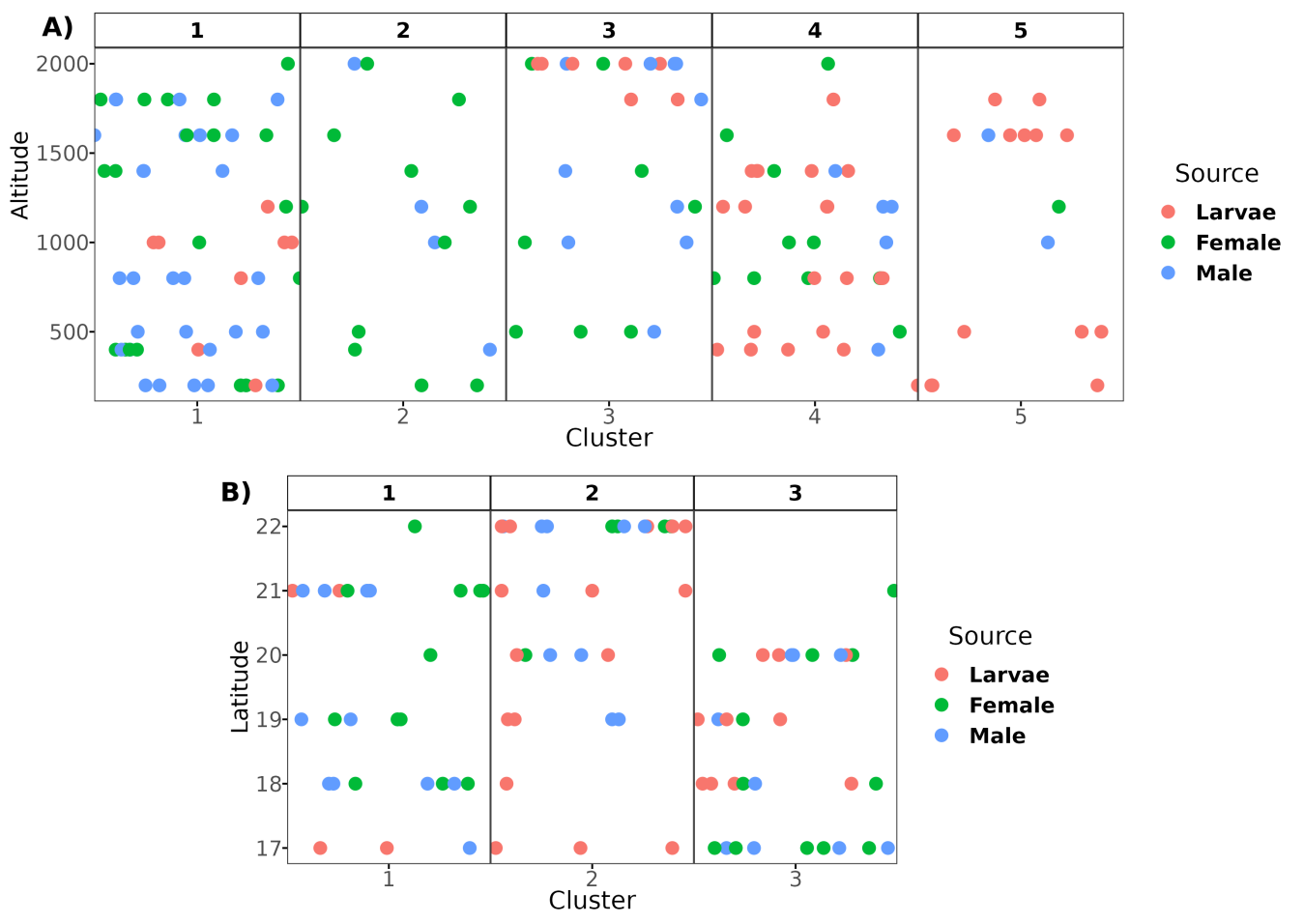


**Figure S1**. Dot plot showing the number and type of samples in each cluster. A) Altitudinal clusters, B) Latitudinal clusters.


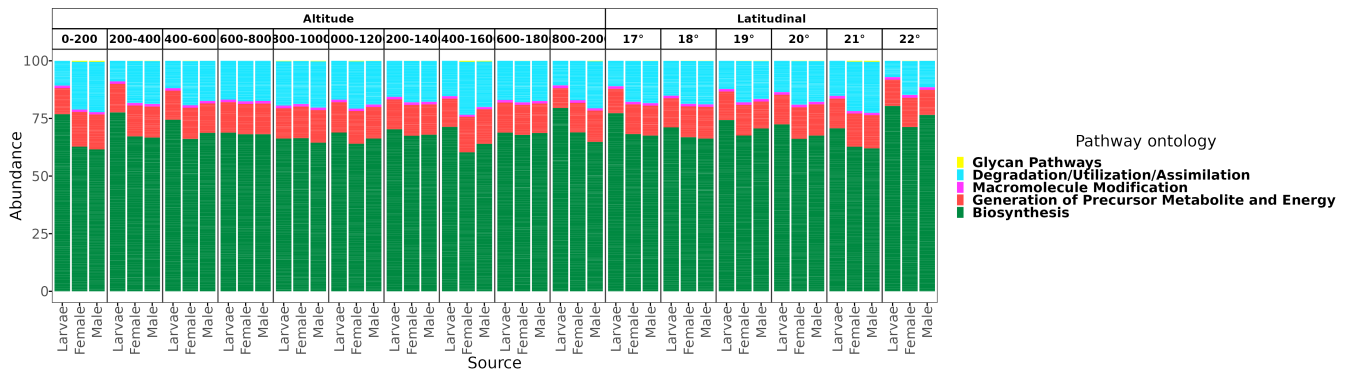


**Figure S2**. Relative abundane of the metabolic pathways predicted with PICRUSt2 classified by the MetaCyc pathway ontology categories.

**Table S1.** Type and number of samples in each altitudinal cluster.

| Cluster | Source | Number of samples | Altitude range |
| --- | --- | --- | --- |
| 1 | \| Larvae \| \| --- \| \| Female \| \| Male \| | \| 8 \| \| --- \| \| 20 \| \| 28 \| | 0-2000 |
| 2 | \| Female \| \| --- \| \| Male \| | \| 11 \| \| --- \| \| 4 \| | 0-600, 800-2000 |
| 3 | \| Larvae \| \| --- \| \| Female \| \| Male \| | \| 7 \| \| --- \| \| 8 \| \| 10 \| | 400-600, 800-1400, 1600-1800 |
| 4 | \| Larvae \| \| --- \| \| Female \| \| Male \| | \| 21 \| \| --- \| \| 10 \| \| 5 \| | 0-2000 |
| 5 | \| Larvae \| \| --- \| \| Female \| \| Male \| | \| 13 \| \| --- \| \| 1 \| \| 2 \| | 0-200, 400-600, 800-1200, 1400-1800 |

**Table S2.** Type and number of samples in each latitudinal cluster.

| Cluster | Source | Number of samples | Latitude range |
| --- | --- | --- | --- |
| 1 | \| Larvae \| \| --- \| \| Female \| \| Male \| | \| 4 \| \| --- \| \| 13 \| \| 11 \| | 17, 18, 19, 20, 21, 22 |
| 2 | \| Larvae \| \| --- \| \| Female \| \| Male \| | \| 16 \| \| --- \| \| 5 \| \| 10 \| | 17, 18, 19, 20, 21, 22 |
| 3 | \| Larvae \| \| --- \| \| Female \| \| Male \| | \| 10 \| \| --- \| \| 12 \| \| 9 \| | 17, 18, 19, 20, 21 |

**Table S3**. Values of the nearest sequenced taxon index (NSTI) calculated by PICRUSt2 on the core ASVs.The Similarity column represent the sequence similarity of the ASV sequence with a given Species determined by the NSTI value.

| ASV ID | NSTI | Similarity | Species |
| --- | --- | --- | --- |
| 0ddf98a2987efff71c8bc2fbf64fa5ba | 0.033166 | 96.6834 | *Achromobacter aegrifaciens* (B) |
| 4d2dc5f5af056a13b46a2695f5b30587 | 0.00015 | 99.985 | *Gluconobacter cerevisiae* (L) (P) |
| 5c302056b95837009851259e229c6528 | 0.00015 | 99.985 | *Acetobacter lambici* (L) (P) |
| 65b901f569c3e1a0a2ba137cf1c8a062 | 0.010946 | 98.9054 | *Enterobacter hormaechei subsp. Xiangfangensis* (A) |
| 74d4069cbc73feae99a305de3cd76023 | 0.000101 | 99.9899 | *Gluconobacter frateurii (*L) |
| 7a76c2a0f7637c8e9168e81fe58a287e | 0.000101 | 99.9899 | *Brucella pseudogrignonensis* (A) |
| 7f605f61282fe9b25a0e900f3e27a081 | 0.010948 | 98.9052 | *Enterobacter mori* (A) |
| a68ace7d7ee4f50ad09513287f7ab7b8 | 0.00015 | 99.985 | *Phyllobacterium myrsinacearum* (B) |
| ae5315becdf1f3033fee7951440de6f9 | 0.135061 | 86.4939 | *Phyllobacterium endophyticum*(L) |
| b4d99952ced36febcddb9f39e9fe2dc8 | 0.00015 | 99.985 | *Acetobacter persici JCM 25330* (L) (P) |
| bcc0353ab537f2ce1577b5479b029610 | 0.000101 | 99.9899 | *Acetobacter suratthaniensis*( L) (P) |
| ea536311f4f59278af9d62817682bbb0 | 0.055351 | 94.4649 | *Acetobacter fabarum* (L) (P) |
